# Clinical characteristics, management, and prognostic outcomes of ipilimumab-induced nephritis

**DOI:** 10.3389/fmed.2025.1693359

**Published:** 2025-12-09

**Authors:** Zhenglin Xiao, Yanfei Xie, Xiang Liu, Ya Liu

**Affiliations:** 1 Department of Clinical Pharmacy, Xiangtan Central Hospital (The Affiliated Hospital Of Hunan University), Xiangtan, China; 2 Department of Pharmacy, People’s Hospital of Ningxiang City, Hunan University of Chinese Medicine, Changsha, China

**Keywords:** ipilimumab, nephritis, acute kidney injury, immune-related adverse events, management

## Abstract

**Background:**

Ipilimumab-induced nephritis is a rare but potentially severe immune-related adverse event with incompletely defined clinical features and outcomes. This study aimed to synthesize its clinical presentation, pathology, management, and prognosis to facilitate timely recognition and evidence-based care.

**Methods:**

We identified cases of ipilimumab-induced nephritis from 28 published articles up to May 31, 2025, and abstracted individual patient-level data for descriptive analysis.

**Results:**

A total of 30 patients were included (26 male, 4 female; median age 63 years [range: 43, 78]). The median onset of nephritis following ipilimumab initiation was 7 weeks (range: 1, 48 weeks). Clinically, patients commonly presented with fatigue (26.7%), fever (20.0%), rash (13.3%), and weight loss (13.3%). Laboratory findings included elevated serum creatinine (median 3.4 mg/dL, range: 1.6, 10.4), eosinophilia (18.5%), proteinuria (14.8%), urinary tract infection (11.1%), increased C-reactive protein (7.4%), and neutrophilic leukocytosis (3.7%). Histopathological analysis revealed acute tubulointerstitial nephritis in 87.5% of cases, followed by IgA nephropathy (12.5%), and interstitial edema (4.2%). The cornerstone of treatment was immediate discontinuation of ipilimumab, coupled with systemic corticosteroids. In select cases, immunosuppressive agents (e.g., mycophenolate mofetil, infliximab, cyclophosphamide) or renal replacement therapies (hemodialysis, plasma exchange) were utilized, though their efficacy requires further validation. Clinical improvement was observed in 93.3% of patients, with a median recovery time of 7 weeks (range: 1, 36 weeks), while 3.3% experienced fatal outcomes.

**Conclusion:**

These findings underscore the importance of early recognition and prompt intervention. A thorough clinical evaluation, including symptom assessment, physical examination, and laboratory testing, is essential for accurate diagnosis. Corticosteroids remain the mainstay of therapy, and early drug withdrawal is critical in mitigating renal injury.

## Introduction

Ipilimumab, a cytotoxic T lymphocyte-associated antigen 4 (CTLA-4) inhibitor, is approved for advanced melanoma and is increasingly used in combination with PD-1 inhibitors such as nivolumab and pembrolizumab across various malignancies, including metastatic renal cell carcinoma (mRCC) and non-small cell lung cancer (NSCLC) (1). While these immune checkpoint inhibitors (ICIs) have significantly improved survival outcomes, their use is associated with a broad spectrum of immune-related adverse events (irAEs) that can affect any organ system (2). Among these, ipilimumab-induced nephritis is a rare but clinically significant complication that may progress from acute kidney injury to chronic renal dysfunction (3). As ipilimumab becomes more widely integrated into cancer treatment protocols, a comprehensive understanding of its renal toxicities, including clinical presentation, diagnostic evaluation, therapeutic strategies, and prognostic implications, is critical for optimizing patient management.

## Methods

### Search strategy

A comprehensive literature search was conducted across English-language databases (PubMed, Embase, and Web of Science) and Chinese-language databases (Wanfang Data and China National Knowledge Infrastructure, CNKI) to identify studies related to ipilimumab-induced nephritis published up to May 31, 2025. The search used a combination of keywords: “Ipilimumab,” OR “anti-CTLA-4,” AND “nephritis,” OR “interstitial nephritis,” OR “tubulointerstitial nephritis,” OR “acute kidney injury,” OR “nephrotoxicity.”

### Inclusion and exclusion criteria

Diagnostic criteria: patients were diagnosed with ipilimumab-induced nephritis if they developed renal impairment, including elevated serum creatinine and/or eosinophilia, or if kidney biopsy revealed acute tubulointerstitial nephritis or other immune-mediated renal abnormalities. Inclusion criteria: clinical studies, case reports, and case series of ipilimumab-induced nephritis. Exclusion criteria: reviews, mechanism studies, animal studies, duplicate cases, articles with insufficient data.

### Data extraction

Two investigators independently performed the initial screening based on predefined eligibility criteria. Discrepancies were resolved through group discussion to reach consensus on study inclusion. Patient-level data were systematically extracted using a custom-designed data collection form, including demographic information (age, sex), medical history, treatment protocols, time to onset, clinical manifestations, laboratory findings, therapeutic interventions, and clinical outcomes.

### Statistical analysis

All statistical analyses were conducted using SPSS version 22.0. Continuous variables were summarized as medians with corresponding ranges (minimum to maximum), while categorical variables were reported as percentages.

## Results

### Basic information

**Figure 1:**
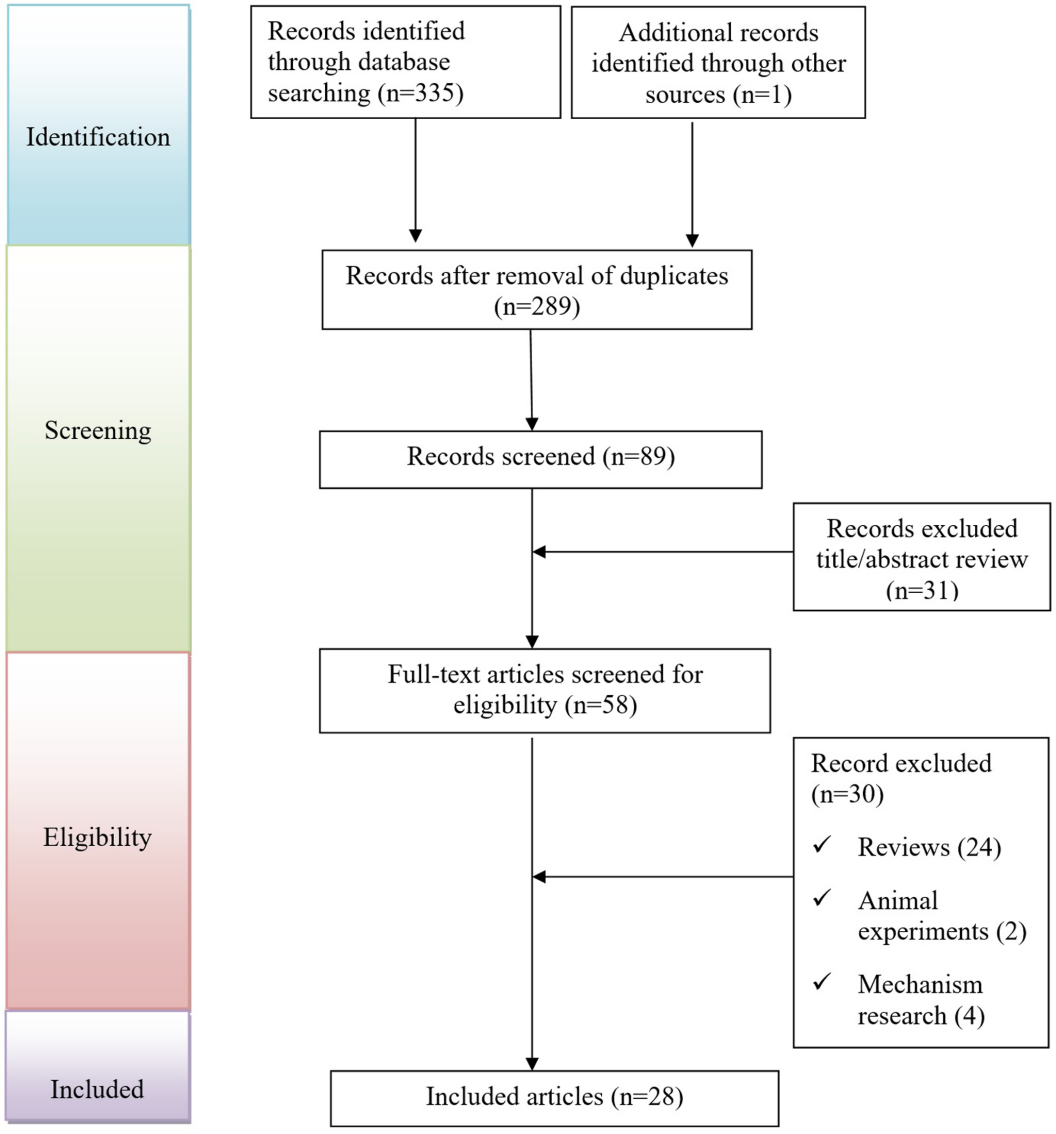
Flow diagram of the selection of studies for inclusion.

As shown in Figure 1, a total of 336 records were initially identified through database searches and manual screening. After removing duplicates and screening titles and abstracts, 28 studies were included for final analysis (4–29). Clinical characteristics of the 30 patients were summarized in Table 1 (29–31). Among the included patients, 26 (86.7%) were male and 4 (13.3%) were female, with a median age of 63 years (range: 43–78). Geographically, most cases were reported from the United States (46.7%), followed by Japan (16.7%), France (13.3%), and other countries including Germany, Mexico, Italy, Canada, and Portugal. The median onset time of nephritis following ipilimumab administration was 7 weeks (range: 1, 48), with 63.3% of cases occurring within the first 10 weeks. The most common treatment indications were melanoma (56.7%) and renal cell carcinoma (30.0%), followed by non-small cell lung cancer (6.7%), Hodgkin lymphoma (3.3%), and malignant pleural mesothelioma (3.3%). Among the patients with available clinical history, 46.7% had underlying conditions such as hypertension, chronic kidney disease, or diabetes. Additionally, 56.7% were receiving concomitant medications, including nivolumab and various supportive agents such as proton pump inhibitors and antihypertensives.

**Table 1:** General data of 30 patients reported in case series/reports.

Parameter	Classification	Value
Gender (30)^a^	Male	26 (86.7%)
	Female	4 (13.3%)
Age (30)^a^	Years	63 (43, 78)^b^
Country (30)^a^	USA	14 (46.7%)
	Japan	5 (16.7%)
	France	4 (13.3%)
	Germany	3 (10%)
	Mexico	1 (3.3%)
	Italy	1 (3.3%)
	Canada	1 (3.3%)
	Portugal	1 (3.3%)
Symptom onset time (30)^a^	Weeks	7 (1, 48)^b^
	1–10	19 (63.3%)
	11–20	7 (23.3%)
	21–30	1 (3.3%)
	31–40	2 (6.7%)
	41–48	1 (3.3%)
Indication (30)^a^	Melanoma	17 (56.7%)
	Renal cell carcinoma	9 (30.0%)
	Non-small cell lung cancer	2 (6.7%)
	Hodgkin lymphoma	1 (3.3%)
	Malignant pleural mesothelioma	1 (3.3%)
Disease history (14)a	Breast cancer, prostate cancer, asthma, hypertension, crescents or interstitial fibrosis, dyslipidemia, chronic kidney disease, end-stage renal disease, diabetes	14 (46.7%)
Concomitant medications (17)^a^	Nivolumab, dabrafenib, trametinib, pantoprazole, amitriptyline, levothyroxine, paracetamol, carvedilol, doxazosin, bezafibrate, lafutidine, ezetimibe, pembrolizumab	17 (56.7%)

^a^Represents the number of patients out of 30 in whom information regarding this particular parameter was provided; ^b^Median (minimum, maximum).

### Clinical manifestations

The clinical characteristics of the 30 patients are summarized in Table 2. The most frequently reported symptoms were fatigue (8 cases, 26.7%), followed by fever (6 cases, 20.0%), rash (4 cases, 13.3%), hematuria (4 cases, 13.3%), and weight loss (4 cases, 13.3%). Additional symptoms included diarrhea (3 cases, 10.0%), anorexia (3 cases, 10.0%), nausea (2 cases, 6.7%), hepatitis (2 cases, 6.7%), and edema (2 cases, 6.7%). Other less common manifestations such as mental status change, arthralgia, abdominal pain, myocarditis, pyuria, blurry vision, eosinophilia, and headache were reported in 8 patients (26.7%). Laboratory data were available for 27 patients. The median serum creatinine level was 3.4 mg/dL (range: 1.6, 10.4). Based on the severity of renal impairment, 14.8% of patients exhibited mild elevation (1.5–2.0 × ULN), 70.3% had moderate impairment (2.0–3.0 × ULN), and 14.8% showed severe elevation (>3.0 × ULN). Eosinophilia was noted in 18.5% of patients, proteinuria in 14.8%, urinary tract infection in 11.1%, while elevated C-reactive protein, neutrophilic leukocytosis, hypercholesterolemia, and hypercalcemia were reported in isolated cases.

**Table 2:** Clinical information of 30 included patients.

Parameter	Clinical features	Value
Clinical symptoms (30)^a^	Fatigue	8 (26.7%)
	Fever	6 (20.0%)
	Rash	4 (13.3%)
	Hematuria	4 (13.3%)
	Weight loss	4 (13.3%)
	Diarrhea	3 (10.0%)
	Anorexia	3 (10.0%)
	Nausea	2 (6.7%)
	Hepatitis	2 (6.7%)
	Edema	2 (6.7%)
	Other symptoms: mental status change, arthralgia, abdominal pain, myocarditis, pyuria, blurry vision, eosinophilia, headache	8 (26.7%)
Laboratory examination (27)^a^	Scr (mg/dL)	3.4 (1.6, 10.4)
	Mild renal impairment: (1.5–2.0) × ULN	4 (14.8%)
	Moderate renal impairmen: (2.0–3.0) × ULN	19 (70.3%)
	Serious renal impairmen: (>3.0) × ULN	4 (14.8%)
	Eosinophilia	5 (18.5%)
	Proteinuria	4 (14.8%)
	Urinary tract infection	3 (11.1%)
	Hypercholesterolemia	1 (3.7%)
	C-reactive protein elevated	2 (7.4%)
	Neutrophilic leukocytosis	1 (3.7%)
	Hypercalcemia	1 (3.3%)
Kidney biopsy (24)^a^	Acute tubulointerstitial nephritis	21 (87.5%)
	IgA nephropathy	3 (12.5%)
	Interstitial edema	1 (4.2%)
	Acute tubular injury/necrosis	1 (4.2%)
	Lupus nephritis	1 (4.2%)

Scr, serum creatinine; ULN, upper limit of normal; IgA, immunoglobulin A.

^a^Represents the number of patients out of 30 in whom information regarding this particular parameter was provided; ^b^Median (minimum, maximum).

### Kidney biopsy

Among 24 patients who underwent kidney biopsy, acute tubulointerstitial nephritis was the predominant lesion (*n* = 21, 87.5%), followed by IgA nephropathy (*n* = 3, 12.5%). Other histological abnormalities, including interstitial edema, acute tubular injury/necrosis, and lupus nephritis, were each identified in a single patient (4.2%).

### Treatment and prognosis

The treatment strategies and clinical outcomes of the 30 patients are summarized in Table 3. All patients discontinued ipilimumab upon the onset of nephritis, and corticosteroids were administered in all cases (100%). Additional immunosuppressive therapies included mycophenolate, infliximab, and cyclophosphamide, each used in one patient (3.3%). Renal support measures were implemented in several cases, including hemodialysis (4 patients, 13.3%), plasma exchange (1 patient, 3.3%), and renal replacement therapy (1 patient, 3.3%). Among the 30 patients, 28 (93.3%) showed clinical improvement, while one patient (3.3%) did not recover and one (3.3%) died. The median time to recovery was 7 weeks (range: 1, 36), with the majority (71.4%) recovering within the first 10 weeks. Notably, two patients experienced disease recurrence following rechallenge with ipilimumab.

**Table 3:** Treatment and prognosis of 30 patients reported in case series/reports.

Parameter	Classification	Value
Treatment (30)^a^	Discontinuation	30 (100%)
	Steroids	30 (100%)
	Mycophenolate	1 (3.3%)
	Infliximab	1 (3.3) ^b^
	Cyclophosphamide	1 (3.3%)
	Hemodialysis	4 (13.3%)
	Plasma exchange	2 (6.7%)
Outcome (30)^a^	Improvement	28 (93.3%)
	No recovery	1 (3.3%)
	Death	1 (3.3%)
Recovery time (28)^a^	Weeks	7 (1, 36)^b^
	1–10	20 (71.4%)
	11–20	6 (21.4%)
	21–30	1 (3.6%)
	31–40	1 (3.6%)
Rechallenge (2)^a^	Relapse	2 (6.7%)

^a^Represents the number of patients out of 30 in whom information regarding this particular parameter was provided; ^b^Median (minimum, maximum).

## Discussion

The introduction of combination ICIs, particularly ipilimumab plus nivolumab, as first-line therapy has markedly improved survival outcomes in patients with advanced clear cell renal cell carcinoma (ccRCC) (25). A recent post-hoc analysis of the Phase III CHECKMATE 214 trial further confirmed the superiority of this regimen over sunitinib, demonstrating a median overall survival (OS) of 31.2 months versus 13.6 months and an objective response rate (ORR) of 57% compared to 19% in patients with mRCC (32). Ipilimumab enhances antitumor immunity by stimulating immune system activation, but it can also disrupt peripheral tolerance to self-antigens, leading to autoimmune responses known as irAEs (24). The underlying mechanisms of irAEs involve cytokine-driven inflammation, antigenic cross-reactivity, and complement-mediated tissue injury.

Drug-induced nephritis is frequently associated with agents such as non-steroidal anti-inflammatory drugs, beta-lactam antibiotics, rifampin, and allopurinol, typically mediated by immune mechanisms involving either cellular or humoral pathways (33). Although relatively uncommon, nephritis related to ICIs represents a clinically significant immune-related adverse event (33). One proposed mechanism implicates drug-specific antibodies that may cross-react with tubular epithelial structures or trigger complement deposition within the renal interstitium (34). Histopathological findings often reveal infiltrates of CD4^+^ and CD8^+^ T cells, supporting the role of T cell–mediated injury. ICIs, by enhancing effector T cell responses, can lead to immune cell infiltration and inflammation in renal tissue (35). Specifically, ipilimumab may precipitate acute interstitial nephritis through T cell activation, while CTLA-4 blockade promotes peripheral T cell expansion, which may inadvertently target non-malignant renal structures and contribute to tissue injury (3).

The onset of ipilimumab-associated nephritis appears to be influenced by a range of clinical and treatment-related factors. Parameters such as elevated body mass index (BMI), underlying autoimmune susceptibility, and therapeutic regimens have been linked to a higher risk of irAEs (36). In this study, 26 of the 30 patients (86.7%) were male, indicating a marked sex imbalance and suggesting that male sex may be a potential risk factor. Most cases occurred in individuals over 43 years old, and the median latency from drug initiation to symptom onset was approximately 10 weeks, indicating a delayed immunologic response. Emerging data also suggest a correlation between prolonged ICI exposure, higher irAE incidence, and sustained treatment efficacy (36). Therefore, for patients receiving extended immunotherapy, treatment strategies should be personalized to balance efficacy and toxicity. Additionally, a notable proportion of patients presented with comorbidities such as hypertension, chronic kidney disease, and diabetes, which may predispose to enhanced renal immune sensitivity (20). Evidence suggests that the risk of kidney injury is significantly increased with combination immune checkpoint blockade (anti-CTLA-4 plus anti-PD-1) compared to monotherapy (22). Moreover, ICIs demonstrate time- and dose-dependent toxicity, with higher drug exposures associated with increased adverse effects (37). In particular, the incidence of ipilimumab-associated nephritis is markedly elevated when used in combination with nivolumab. This dual immune checkpoint inhibition strategy has been associated with both a greater frequency and earlier onset of renal immune-related adverse events, including nephritis (38). These observations highlight the critical importance of close renal function surveillance during combination immunotherapy.

Ipilimumab exhibits a terminal half-life of approximately 15.4 days, with steady-state concentrations typically reached after multiple dosing cycles. As a fully human IgG1 monoclonal antibody targeting CTLA-4, it is primarily cleared via non-specific proteolytic catabolism (38). Previous studies have shown that ipilimumab is associated with a broad spectrum of irAEs, including dermatitis, colitis, hepatitis, and endocrinopathies (1). In our analysis of 30 patients, the most frequently reported clinical manifestations were fatigue, followed by fever, rash, hematuria, weight loss, and hepatitis. Renal function testing revealed a median serum creatinine level of 3.4 mg/dL (range: 1.6, 10.4), typically more than double the upper limit of normal, reflecting significant renal impairment. Additional laboratory abnormalities included eosinophilia, proteinuria, urinary tract infections, elevated C-reactive protein, neutrophilic leukocytosis, and hypercholesterolemia, consistent with systemic immune activation and renal involvement. Kidney biopsy findings predominantly showed acute tubulointerstitial nephritis, IgA nephropathy, interstitial edema, acute tubular injury/necrosis, and lupus nephritis, further confirming immune-mediated renal injury as the underlying pathology.

Effective management of ipilimumab-induced nephritis requires prompt drug discontinuation, followed by initiation of systemic corticosteroid therapy. Premature cessation or insufficient dosing of corticosteroids may result in incomplete resolution and progression to secondary renal impairment. Most patients experience clinical improvement, with a median recovery time of approximately 7 weeks. However, cessation of ipilimumab may compromise tumor control. Lemoine et al. reported a case in which steroid therapy achieved partial renal remission, but disease progression occurred in the absence of effective alternative treatment for advanced melanoma (5). This highlights the clinical dilemma of whether ICIs can be safely resumed or continued in patients who develop nephrotoxicity.

It is noteworthy that the cases included in this study were from diverse geographic regions and clinical settings, which may introduce variability in diagnostic and treatment practices, thus affecting the clinical presentation and management of ipilimumab-induced nephritis. This represents a limitation inherent in case-based studies. Nevertheless, the consistency observed in key clinical features (such as common symptoms, laboratory markers, and histopathological findings) and treatment responses (such as clinical improvement following corticosteroid therapy) strengthens the reliability of our conclusions. These findings offer valuable insights for the clinical management of ipilimumab-induced nephritis. Future research should focus on developing guidelines for the management of immune-related nephritis, with standardized diagnostic and treatment protocols to minimize variability and improve clinical guidance.

### Limitations of the study

Several limitations should be acknowledged. First, the small sample size of 30 cases may limit the generalizability of the findings and may result in selection bias. Second, incomplete clinical information in some reports, including the absence of histopathological data in certain patients, may reduce the accuracy of the analysis. Third, additional prospective studies and randomized controlled trials are required to clarify the clinical features and underlying mechanisms of ipilimumab-induced nephritis. Nonetheless, the present findings offer valuable clinical implications that may support clinicians in recognizing and managing similar immune-related renal complications.

## Conclusion

Ipilimumab-induced nephritis is a rare but potentially severe complication of immune checkpoint therapy. Early diagnosis and treatment with corticosteroids are essential for improving renal outcomes. While most patients recover, some may experience chronic kidney dysfunction, emphasizing the need for close monitoring and individualized care. Future studies should focus on identifying predictive biomarkers for nephritis and developing more effective management strategies.

## Data Availability

The original contributions presented in the study are included in the article/supplementary material, further inquiries can be directed to the corresponding authors.
